# Extracellular Vesicles as Drug Delivery System for Cancer Therapy

**DOI:** 10.3390/pharmaceutics16081029

**Published:** 2024-08-01

**Authors:** Jin Wang, Bohang Yin, Jiabing Lian, Xia Wang

**Affiliations:** 1 School of Life Sciences, Liaoning University, Shenyang 110036, China; wangjin@lnu.edu.cn (J.W.); 15374969118@163.com (J.L.); 2 Department of Surgical Oncology and General Surgery, The First Hospital of China Medical University, Shenyang 110001, China; yinbohang9909@163.com; 3 Institute of Health Sciences, China Medical University, 77 Puhe Road, Shenyang 110122, China

**Keywords:** extracellular vesicles, drug delivery, cancer therapy, engineering

## Abstract

In recent decades, the pursuit of drug delivery systems has led to the development of numerous synthetic options aimed at enhancing drug efficacy while minimizing side effects. However, the practical application of these systems is often hindered by challenges such as inefficiency, cytotoxicity, and immunogenicity. Extracellular vesicles, natural carriers for drugs, emerge as promising alternatives with distinct advantages over synthetic carriers. Notably, EVs exhibit biocompatibility, low immunogenicity, and inherent tissue-targeting capabilities, thus opening new avenues for drug delivery strategies. This review provides an overview of EVs, including their biogenesis and absorption mechanisms. Additionally, we explore the current research efforts focusing on harnessing their potential as drug carriers, encompassing aspects such as purification techniques, drug loading, and bioengineering for targeted delivery. Finally, we discuss the existing challenges and future prospects of EVs as therapeutic agents in clinical settings. This comprehensive analysis aims to shed light on the potential of EVs as versatile and effective tools for drug delivery, particularly in the realm of cancer therapy.

## 1. Introduction

The development of drug delivery systems plays a crucial role in facilitating the controlled release of pharmaceutical ingredients to achieve desired therapeutic outcomes. These technologies have significantly enhanced treatment efficacy across various medical domains, ranging from improving therapeutic efficacy and reducing toxicity to enabling novel treatment modalities and enhancing patient compliance [1]. In recent years, the development of synthetic nanoparticulate delivery systems has garnered considerable attention for their potential to augment the pharmacokinetic and pharmacodynamic profiles of therapeutic agents, particularly in the context of cancer therapy [2,3]. Lipid-based nanocarriers, among these delivery systems, offer a versatile platform for drug encapsulation, leading to the clinical translation of several formulations [4]. Capitalizing on the enhanced permeability and retention effect (EPR) and leveraging antigens specific to tumor cells, these nanocarriers have shown promise in increasing intracellular drug concentrations while minimizing toxicity in non-tumor cells [5,6,7]. However, despite these advancements, challenges such as toxicity, low biocompatibility, and off-target effects persist due to physicochemical factors and complex compositions, and it is thus necessary to develop more biocompatible alternatives in addition to synthetic nanocarriers [8,9,10,11]. In light of these challenges, extracellular vesicle-based carrier systems have garnered significant attention.

Extracellular vesicles (EVs) play pivotal roles in various physiological and pathological processes by facilitating intercellular communications [12]. These nanoscale lipid bilayer vesicles, actively secreted by most cell types, serve as natural vehicles for transporting bioactive molecules such as nucleic acids, proteins, lipids, and metabolites [13]. EVs present compelling advantages as drug delivery systems. Their stable membrane structure confers protection to cargo molecules in circulation, enabling long-term delivery of therapeutics while evading immune surveillance [14,15,16]. Moreover, EVs exhibit remarkable tissue-penetrating abilities, including the capacity to traverse the blood-brain barrier (BBB), a significant hurdle for conventional therapeutics [17,18]. Their natural targeting capabilities enable specific binding to tumor cells, thereby enhancing drug delivery efficacy while minimizing off-target effects on normal tissues. Overall, the advantages of EVs include low immunogenicity, non-toxicity, excellent biocompatibility, and the ability to penetrate inaccessible tissues, all of which have sparked interest in leveraging EVs as next-generation drug delivery platforms.

EVs have emerged as a highly promising system for cancer treatment, with extensive research exploring their use in delivering a variety of anti-tumor therapeutic molecules. Several of these studies have advanced to preclinical trial stages [19], highlighting the potential of EVs in this field. Utilizing EVs as drug carriers involves carefully selecting their sources and establishing robust protocols for isolation, therapeutic molecule loading, and potential modifications to generate suitable nanocarriers. Significant progress has been made in introducing exogenous ingredients into EVs. Their versatility includes loading various therapeutics, such as small molecules, RNAs, and proteins, via bioengineering techniques. Effective delivery of bioactive molecules via EVs requires precise targeting of specific recipient regions. By enriching characteristic surface proteins, EVs can be adapted with targeting components and other modifications to enhance their functionality. Typically, EVs are engineered to increase their affinity for target cells and extend their circulation time, optimizing drug delivery and enhancing concentration within the tumor microenvironment [20]. This strategic combination of cargo loading and surface modification establishes engineered EVs as a promising platform for precision therapy [20,21].

Despite extensive research, the clinical application of EV-based therapies faces numerous challenges, such as selecting suitable EV sources, establishing methods for efficient cargo loading, and accurately directing EVs toward specific target regions. EVs have been sourced from a diverse array of origins, including various mammalian cells, tumor cells, plant cells, and body fluids [22]. The characteristics inherited from their parent cells may significantly influence EV functionality. It has been shown that EVs isolated from different cell types, like mesenchymal stem cells, T cells, and platelets, exhibit unique molecular patterns and have distinct effects on their cellular targets due to their varied contents [23]. To improve treatment outcomes and minimize potential side effects, it is crucial to select appropriate EV sources that match the therapeutic targets and enhance the efficiency of therapeutic cargo loading. Selected cargos are encapsulated into EVs using various in vitro physicochemical techniques following isolation or through in vivo bioengineering strategies to modify the parent cells. However, employing EVs as clinical drug delivery platforms faces technical challenges, primarily due to the lack of standardization in the development of drug-loaded EVs.

Research on EVs as drug delivery systems is advancing rapidly. A systematic understanding of the strategies for sourcing, purification, characterization, cargo loading, and modification is crucial to effectively utilize EVs in cancer therapy. This review begins by briefly describing the history, biogenesis, and uptake of the main EV subtypes. It then provides a comprehensive overview of current methodologies for harnessing EVs as efficient drug delivery systems in cancer treatment, with a focus on general drug loading techniques and advanced surface modifications with exogenous surface targeting ligands to enhance the in vivo circulation and molecular targeting capabilities of EVs. Several representative studies on EV-based anti-cancer therapies are discussed to highlight the progress and challenges in employing EVs as versatile drug carriers. By highlighting recent innovations in the engineering of EVs for targeted delivery of therapeutic agents, particularly in cancer treatment, the review aims to enhance understanding of the current research landscape and future directions for EV-based strategies in targeted drug delivery.

## 2. Discovery and Development of Extracellular Vesicles

### 2.1. Discovery of Extracellular Vesicles

**Figure 1:**
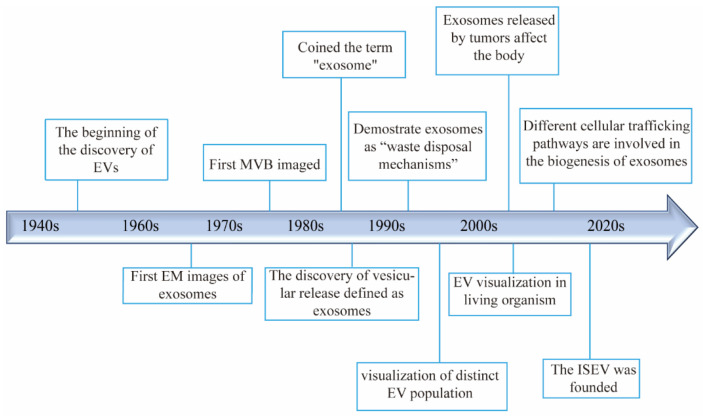
Timeline and milestones in the research of extracellular vesicles.

EVs have been recognized as important biological entities for over 50 years (Figure 1). In 1946, Chargaff and West identified a thrombin-like coagulation factor while studying anemia, marking the nascent stage of EV biology [24]. Later, Peter Wolf published electron microscope images of these particles [25]. In 1983, Johnstone et al. observed the release of transferrin metabolites in the form of small vesicles during the maturation of sheep reticulocytes [26,27]. In the 1990s, Johnstone described exosomes as a “waste disposal mechanism” with enzymatic activity in reticulocytes [28]. Additionally, studies began linking exosome abundance with various diseases, broadening EV research in diagnostics and therapeutics [29,30]. Subsequently, small vesicles were identified from various sources, including blood, urine, ascites, synovial fluid, and saliva in humans, as well as in animals, plants, bacteria, fungi, and parasites [31,32,33,34]. The International Society for Extracellular Vesicles (ISEV) recommended “extracellular vesicles (EVs)” as a universal term for these vesicular structures [35]. Further research revealed that EVs facilitate intercellular communication by delivering molecular substances, playing roles in numerous physiological and pathological processes across diverse cell types [36].

### 2.2. Biogenesis and Classification of Extracellular Vesicles

EVs are a diverse group of vesicular entities ranging in diameter from 30 to 5000 nm [37]. Different types of EVs have been classified based on their different origin, size, and biogenesis. At least three major EV modes are known: exosomes, ectosomes, and apoptotic bodies.

*Exosomes* are the most-studied type of relatively small EVs with diameters ranging from 30 to 150 nm [38]. They are primarily produced via the endocytic endosomal pathway [37]. The cytoplasmic membrane buds inward, leading to the capture of membrane molecules and the formation of early endosomes within the cell [39]. Early endosomes formed by the fusion of early endocytic vesicles mature into late endosomes, which then invaginate to form intraluminal vesicles (ILVs) before developing into multivesicular bodies (MVBs) containing various cargo molecules [40]. Subsequently, a fusion of MVBs with the plasma membrane results in ILVs being released to the extracellular space [41]. The endosomal sorting complex required for transport (ESCRT) machinery is important for sorting proteins to ILVs in the MVBs. ESCRT-accessory proteins like TSG101, ALIX, and VPS4 are important for the outward budding of the plasma membrane in exosome formation. Many of the tetraspanin proteins, including CD63, CD81, and CD9, are highly enriched in exosomes and have long been used as exosome marker proteins, although some small EVs containing these proteins may bud directly from the plasma membrane [42,43]. The RAB family proteins play a significant role in vesicle trafficking and exosome release. Specific RAB proteins (RAB7, RAB11, RAB35, RAB27A, RAB27B) regulate various stages of exosome secretion [44]. Therefore, inhibition of these proteins could make disruption of normal exosome secretion. Further research is needed to fully understand the roles of RAB proteins in the biogenesis of exosomes and other EV types.

*Ectosomes*, ranging in size from 100 to 1000 nm, are directly formed by the detachment of the plasma membrane through outward budding [43]. Ectosomes usually comprise diverse types of EVs, such as microvesicles and oncosomes. The classical microvesicles (150 to 1000 nm) are characterized by annexin A1 and A2 expression and lower flotation densities compared to small EVs [45,46,47]. There is also small arrestin domain-containing protein 1 (ARRDC1)-mediated microvesicles, characterized by ARRDC1 and TSG101 expression and requiring VPS4 activity [48]. T cells can release ~70 nm synaptic ectosomes at the immunological synapse, dependent on TSG101 for sorting of T cell receptors and VPS4 for vesicle scission [49]. Perivascular dendritic cells could release 500–1000 nm ectosomes to induce anaphylaxis by relaying allergens to mast cells [50]. Cells can also release tetraspanin (CD9, CD63, CD81) positive exosome-sized small ectosomes directly from the plasma membrane [51]. The formation of microvesicles requires actin cytoskeleton rearrangement for plasma membrane budding, scission, and vesicle release. The small GTP-binding protein ADP ribosylation factor 6 (ARF6) is a critical regulator of classical microvesicle biogenesis, activating phospholipase D to recruit ERK, which further activates myosin light chain kinase (MLCK) to phosphorylate MLC for actin cytoskeleton contraction [52]. ARF6 was also discovered to play important roles in loading pre-miRNA and DNA cargo to microvesicles shed from tumor cells [53,54]. Actually, ARF6 was revealed to play important roles in the biogenesis of multiple EV types [42,55]. Oncosomes, 1–5 μm microvesicles released from tumor cells due to oncoprotein overexpression, also characterized by ARF6 and annexin A1 expression [56,57].

*Apoptotic bodies*, ranging in size from 1000 to 5000 nm, are membrane-blebbing protrusions formed from apoptotic cells during the process of programmed cell death [58,59]. It is well known that apoptotic bodies facilitate the clearance of damaged cellular debris, ultimately being removed through phagocytosis [60]. Therefore, apoptotic bodies are characterized by the presence of histones, DNA, and proteins of nuclear, ER, and mitochondrial origin. The formation of apoptotic bodies can effectively prevent the impact of toxins and degradative enzymes on cellular integrity [61]. Recent studies indicated that apoptotic bodies could function in immune regulation and inflammation within the tumor microenvironment during the early stages of apoptosis [60,62]. Phosphatidylserine (PS) is translocated to the outer leaflet during apoptosis, acting as an “eat me signal” for phagocytic clearance. Annexin V is commonly used to detect apoptotic cells and EVs by binding to outer leaflet PS [63]. Small apoptotic EVs expressing annexin V typically exhibit low levels of CD63, CD81, CD9, ALIX, and TSG101 [64], indicating a pathway separate from classical exosomes.

In addition to the previously outlined three subtypes of EVs, emerging subpopulations such as migrasomes, exophers, and autophagy-related EVs were recently characterized [44]. However, exosomes stand out prominently as a primary choice for drug delivery due to their relatively uniform small size, stability, biocompatibility, and well-defined biogenesis process.

### 2.3. Absorption/Uptake of Extracellular Vesicles

EVs carry a diverse array of cargo capable of significantly influencing the phenotype of recipient cells. For effective drug delivery to target cells, EVs must undergo fusion with cell membranes, either directly with the plasma membrane or subsequent to endocytic uptake into endosomes [65]. The process of EV uptake typically encompasses several stages, including recipient cell recognition, internalization, cargo release, and intracellular processing. Herein, we provide an overview of the principal routes involved in EV cellular uptake.

Exosomes are primarily internalized via macropinocytosis and clathrin-independent endocytosis pathways, involving the coordinated action of various key molecules [66,67,68,69]. Macropinocytosis relies on tyrosine kinase activity, while clathrin-independent endocytosis necessitates the function of Na^+^/H^+^ exchangers and phosphoinositide 3-kinase [67]. Surface proteins on exosomes, notably Tetraspanins, facilitate their uptake by interacting with corresponding receptors on target cells and are implicated in cell-cell fusion events [70,71], thus underpinning the potential application of exosomes in drug delivery.

Microvesicle uptake is closely associated with cholesterol-rich membrane domains such as lipid rafts and caveolae, which provide a platform for interaction with specific membrane proteins on target cells, thereby enhancing internalization [72]. Small GTPases, including RhoA and Rac1, modulate the interaction between microvesicles and the target cell membrane, as well as the endocytic process by regulating cytoskeletal reorganization [73,74,75]. Importantly, these GTPases can influence the dynamics of membrane domains linked to lipid rafts, further facilitating efficient microvesicle internalization.

Apoptotic body uptake relies on surface recognition and internalization mechanisms mediated by specific receptors [76]. PS exposed on apoptotic bodies serves as an “eat me” signal, recognized by receptors such as TIM (T-cell immunoglobulin and mucin domain) family proteins and Bai1 (brain-specific angiogenesis inhibitor 1) on healthy cells [60,77]. The engagement of these receptors ensures effective processing and recycling of proteins, nucleic acids, and other contents of apoptotic bodies, thus laying the groundwork for drug delivery applications.

Overall, cells may employ a diverse array of endocytic pathways, including clathrin-dependent and -independent mechanisms such as caveolin-mediated uptake, macropinocytosis, phagocytosis, and lipid raft-mediated internalization, for EV uptake. Given the heterogeneous nature of EV populations, cells may utilize multiple entry routes. Understanding the precise mechanisms governing EV uptake is crucial for advancing translational studies utilizing EVs as carriers in drug delivery systems, enabling the transport of functional cargos to specific target cells with high precision in biodistribution and minimal immunogenicity.

## 3. Strategies for Utilizing Extracellular Vesicles as a Drug Delivery System

To effectively utilize EVs as drug delivery vehicles for cancer therapy, it is crucial to optimize strategies that encompass the sourcing, purification, characterization, and loading of therapeutic cargo into these vesicles. We provide a comprehensive overview of the current approaches in harnessing EVs as efficient drug delivery systems for cancer therapy, with the aim to provide insights into the advancements and challenges in utilizing EVs as versatile carriers for cancer therapy.

### 3.1. Sources of Extracellular Vesicles for Drug Delivery

EVs represent a diverse array of potential drug delivery vehicles sourced from various cell types, including but not limited to mesenchymal stem cells, immune cells, and tumor cells [78,79,80]. However, it is essential to acknowledge that EVs may retain similar content and surface proteins, which reflect the characteristics of their parent cells, influencing their functionality [81]. Additionally, the biodistribution of EVs may be influenced by their origin and route of administration, with certain cell types known to secrete EVs more abundantly than others [82,83,84]. Consequently, careful consideration must be given to selecting the most appropriate sources for isolating EVs to ensure the efficacy and specificity of drug delivery in cancer therapy research.

*Mesenchymal stem cells* EVs released by stem cells play a role in maintaining the survival and pluripotency of these cells. Mesenchymal stem cells (MSCs), adult stem cells predominantly found in bone marrow and adipose tissue, are known for their robust immunomodulatory and regenerative capabilities [85,86,87]. MSCs have been shown to possess the ability to migrate to tumors and sites of inflammation while displaying intrinsic therapeutic properties [88]. Therefore, they are considered to be a promising source for the production of EVs. Among the therapeutic cargo carried by MSC-derived EVs (MSC-EVs), TNF-related apoptosis-inducing ligand (TRAIL) is a membrane protein capable of selectively inducing apoptosis in cancer cells. Research demonstrated that TRAIL expressed in MSC-EVs exhibits significant cytotoxic effects in lung and breast cancer cells, including those resistant to TRAIL [89]. In addition, MSCs-EVs loaded with small molecular therapeutics such as paclitaxel (PTX), doxorubicin (DOX), and gemcitabine have also shown remarkable efficacy in inhibiting the proliferation of various cancer cell types [78,90,91,92,93]. These findings underscore the potential of MSC-derived EVs as effective drug-delivery vehicles for cancer therapy.

*Immune cells* Immune cells serve as pivotal components of the body’s defense mechanism and are extensively utilized for the production of EVs. These EVs derived from immune cells play instrumental roles in modulating cancer immune responses [94,95]. Macrophages-derived EVs loaded with therapeutics, including doxorubicin (DOX), paclitaxel (PTX), and gemcitabine, have exhibited significant inhibitory effects on various cancer types such as ovarian, prostate, pancreatic, lung, and breast cancer [84,96,97,98]. Moreover, dendritic cell-derived EVs, particularly those incorporating the iRGD targeting peptide, have shown promise in pre-clinical models of breast cancer treatment. Tian et al. illustrated that EVs carrying doxorubicin (DOX) induce growth inhibition of breast cancer cells both in vitro and in vivo [99]. In addition to loading small molecular therapeutics, EVs derived from natural killer cells (NK-EVs) possess cytotoxic proteins such as perforin, granzyme A, granzyme B, granulysin, and Fas ligand, which contribute to cancer cell death [100]. Furthermore, the naive properties of NK-EVs secreted from cells exposed to interleukin 15 can be further augmented to enhance tumor targeting ability and cytotoxicity across various cancers [101]. These findings suggest EVs derived from immune cells are pivotal players in orchestrating and modulating immune responses and serve as versatile and effective platforms for targeted cancer therapy.

*Tumor cells* EVs derived from cancer cells, known for their high production rates and specific tumor-homing capabilities, present a competitive edge as drug delivery carriers for targeted chemotherapy [102]. Research has shown that EVs loaded with chemotherapy drugs can significantly enhance drug efficacy, substantially reducing tumor burden [103]. For example, EVs derived from pancreatic cancer cells, loaded with paclitaxel (PTX) or gemcitabine, have been shown to accumulate effectively at tumor sites, exerting anti-tumor effects with minimal damage to normal tissues [98]. Similarly, EVs derived from A549 lung cancer cells containing platinum have demonstrated effective therapeutic outcomes in patients with platinum-resistant advanced lung cancer, significantly reducing the overall tumor cell burden [103,104,105]. Moreover, EVs derived from various tumor cells, including breast cancer cells, ovarian cancer cells, prostate cancer cells, colon cancer cells, and glioblastoma cells, have been developed to load small molecule therapeutics, showing remarkable efficacy in cancer treatment [80,106,107,108,109,110,111].

However, cancer cell-derived EVs can also influence tumor progression by activating several pathological pathways and exerting immunosuppressive effects. The use of these EVs for drug delivery might introduce endogenous cargo molecules that activate pathological pathways [112,113]. The main advantage of using cancer cell-derived EVs for therapeutic purposes is the presence of tumor-specific antigens, which can prime immune cells to induce an immune response. Loading EVs with inhibitors of immunosuppressive cells or immunostimulatory compounds can effectively counteract the immunosuppressive response and enhance anti-tumor immune effects [114,115]. For example, an exosome-based tumor antigen-adjuvant co-delivery system from melanoma has been shown to effectively induce tumor antigen-specific immune responses and inhibit melanoma tumor growth in vivo [116].

In conclusion, cancer cell-derived EVs exhibit promising potential as delivery carriers for therapeutics and co-delivery of immune-stimulating adjuvants, eliciting potent anti-tumor effects. However, it is crucial to clarify and reduce harmful EV contents to minimize potential side effects.

*Other cell lines* Several other commonly used cell lines in the laboratory also serve as sources for the production of EVs as drug carriers [117]. Notably, these include human embryonic kidney 293 cells (HEK293), Chinese hamster ovary cells (CHO), and human embryonic stem cells [118]. The HEK293 cell line is particularly prominent due to its extensive use in EV-mediated drug delivery and its significant potential for industrial applications. EVs derived from HEK293 cells are notable for their high transfection efficiency and their capacity to be easily loaded with various bioactive molecules, such as different RNAs and proteins [119,120]. Furthermore, HEK293 cells can be readily genetically engineered to produce EVs tailored for clinical applications, enhancing their suitability for therapeutic use.

Overall, the choice of cell line significantly influences the characteristics of the derived EVs, including their cargo, targeting capabilities, and therapeutic efficacy. As research progresses, optimizing the selection of cell lines for EV production will be crucial for developing effective and scalable EV-based therapies for cancer and other diseases.

*Bodily fluids* EVs can be extracted from numerous bodily fluids, including urine, plasma, milk, ascites, cerebrospinal fluid, and semen [121]. Red blood cells (RBCs) have been used safely and routinely for blood transfusions over decades. RBCs can be used as universal donors for large-scale EV production. RBC-derived EVs for the delivery of RNA drugs showed robust microRNA knockdown and gene knockout with CRISPR-Cas9 genome editing in leukemia and breast cancer cells [122]. Similarly, doxorubicin (DOX) or sorafenib-loaded EVs from RBCs exhibited significant therapeutic effects on hepatocellular carcinoma [123]. Bovine milk has been discovered to contain a notably high abundance of separable EVs. These bovine milk-derived EVs are regarded as excellent carriers for a variety of therapeutic agents, such as withaferin A, doxorubicin (DOX), anthocyanidins, curcumin, docetaxel, and paclitaxel (PTX) [124,125,126]. Importantly, experiments demonstrated that the EVs from milk did not induce cytotoxicity or allergic reactions [127]. The bioactive molecules carried by bovine milk exosomes remain stable under the harsh conditions of the gastrointestinal tract, thereby supporting their use in oral drug delivery [126,128]. Consequently, milk-derived EVs are emerging as one of the most promising vectors with high stability and low immunogenicity for drug delivery in clinical applications.

*Other sources* EVs derived from plants and bacteria have garnered significant attention for their potential as drug delivery carriers. For example, edible ginger-derived EVs loaded with doxorubicin (DOX) demonstrated excellent tissue compatibility and anti-tumor effects in colorectal cancer [129]. Similarly, grapefruit-derived EVs were shown to effectively deliver various therapeutic drugs and enhance their ability to target inflammatory tumor sites [130]. Bacterial outer membrane vesicles or protoplast-derived EVs loaded with therapeutics have been found to enhance anti-tumor effects [131,132]. These findings highlight the potential of plant- and bacteria-derived EVs as innovative drug delivery systems for cancer therapy.

### 3.2. Characterization and Purification of Extracellular Vesicles

#### 3.2.1. Characterization of Extracellular Vesicles

The characterization of purified EVs is a prerequisite for their use as safe and efficient drug delivery vehicles. The ISEV has developed and periodically updated the Minimal Information for Studies of Extracellular Vesicles (MISEV) guidelines for EV research and applications [35,133]. Key recommendations for characterization are summarized as follows: (1) Quantitative measures of the EV source should be utilized to determine each EV preparation (for example, number of secreting cells, volume of biofluid, and tissue mass). (2) Estimates of EV abundance should be made, including particle count, protein, and/or lipid content. (3) EV preparations should be analyzed for the presence of components specific to EV subtypes or EVs in general, based on the desired specificity. (4) Determine the amount of non-vesicular, co-isolated components present. (5) Indicate the instrument/method limit of detection (LOD) when EVs are defined using quantitative measurements. Further details about several approaches to EV characterization can be found in MISEV (2018, 2020, 2023) [35,133,134]. General characterization of EVs includes assessing particle quantity, size distribution, morphology, and contents to verify the results of separation methods and ensure the quality of purified EVs meets the standards required for clinical applications [135]. A comprehensive characterization of EVs often necessitates the use of multiple techniques. Nanoparticle tracking analysis is employed to determine the particle quantity and size distribution of EVs by analyzing scattered light and the Brownian motion of vesicles. Flow cytometry, transmission electron microscopy, and atomic force microscopy are often used to monitor the size distribution, morphology, and structure of EVs. Additionally, combining the aforementioned physical detection methods with biochemical techniques, including immunofluorescence, immunogold, nucleic acid hybridization, western blot, and ELISA, etc., enables further qualitative and quantitative analysis of the components carried by EVs [19]. Moreover, advanced omics analysis methods such as RNA sequencing (transcriptomics) and mass spectrometry (proteomics and lipidomics) analysis can further be applied to provide a detailed composition of drug-loaded EVs, enhancing their potential for clinical application [136]. By employing these diverse and complementary techniques, researchers can ensure that EVs are thoroughly characterized, providing crucial insights into their functionality and therapeutic potential. This multi-faceted approach is essential for the development of EVs as reliable and effective drug delivery systems for cancer therapy.

#### 3.2.2. Purification of Extracellular Vesicles

EVs are usually isolated from culture mixtures or highly complex biological samples, as mentioned above, unavoidably containing several contaminants such as cellular debris, other extracellular particles, nuclear acids, secreted proteins, etc. Proper purification techniques are essential to obtain EVs that are free from these contaminants, which may interfere with their therapeutic function in clinical application. Currently, various isolation methods have been developed based on the physical and chemical features of EVs, such as centrifugation-based methods, size exclusion methods, immune-affinity capture, polymer precipitation-based methods, etc. [137].

*Centrifugation* Centrifugation-based methods for the purification of EVs can usually be divided into ultracentrifugation and density gradient centrifugation. Differential ultracentrifugation is the most basic and commonly used purification technique, which separates most contaminants and EVs through centrifugation at different speeds. While this method is simple and effective for handling large-scale samples, ultracentrifugation can be time-consuming, have low EV yield, result in poor integrity or aggregation, and may lead to the co-purification of other non-EV contaminants [137]. Density gradient centrifugation can achieve a high yield and purity of EVs. It employs a medium of specific density, such as sucrose strontium iodide, to create a density gradient, which allows vesicles of different densities to stratify within the gradient, thereby isolating EVs with higher purity [138]. However, this method is labor-intensive, time-consuming, and not suitable for high-throughput applications. Similar to differential ultracentrifugation, EVs may show impaired functionality or form aggregates during centrifugation at high speed [139,140].

*Size-exclusion chromatography (SEC)* SEC offers an alternative and increasingly popular approach by separating EVs based on their size. It can produce a high-yield isolation while preserving the biophysical and functional properties of the isolated vesicles [141,142]. In this method, samples are passed through a column filled with stationary phases that allow smaller molecules to enter and be retained longer, while larger EVs pass through more quickly. By combination with ultracentrifugation and/or ultrafiltration, the SEC method could isolate EVs with significantly improved purity and a relatively low cost [141,143]. SEC is advantageous because it can process large volumes and maintain the integrity of EVs, but it may not completely eliminate similarly sized contaminants.

*Immunoaffinity purification* These techniques usually utilize specific antibodies that bind to EV surface-specific proteins, such as CD63 and CD9, enabling the selective isolation of EVs [144]. This method involves incubating the mixture with antibody-coated beads or columns, allowing EVs to bind to the antibodies, and subsequently washing away unbound materials [54]. Immunoaffinity capture can achieve high specificity and purity EVs, but it is limited by the availability of suitable antibodies, can be costly, and yields low quantities of EVs.

*Precipitation-based methods* In precipitation-based methods, the relevant sample mixture containing EVs was incubated with the designed chemical solutions, followed by centrifugation to separate EVs [145]. Polymer- or charge-based precipitation has been successfully used to isolate EVs from biofluid samples. For example, polyethylene glycol (PEG), cationic fish sperm proteins, and ammonium sulfate were applied to isolate EVs from cell culture supernatant body fluid [41]. These precipitation-based methods are simple and do not need to use complex equipment. Several commercial kits based on polymer co-precipitation, such as Exoquick^®^, PureExo^®^, or miRCURY™, are available for EV isolation. However, the purity of the isolated EVs is typically low due to the lack of selectivity in these procedures.

In summary, the purification of EVs is a multi-step process that requires careful selection and optimization of techniques to ensure high purity and functionality. Many other promising methods, such as anion exchange/hydrophobic chromatography microfluidic, have also been developed for isolating EVs [135]. Every purification technique has advantages and disadvantages, and integrating different purification methods seems to pave the way for further development of EVs in therapeutic applications.

### 3.3. General Methods for Drug Loading into Extracellular Vesicles

**Figure 2:**
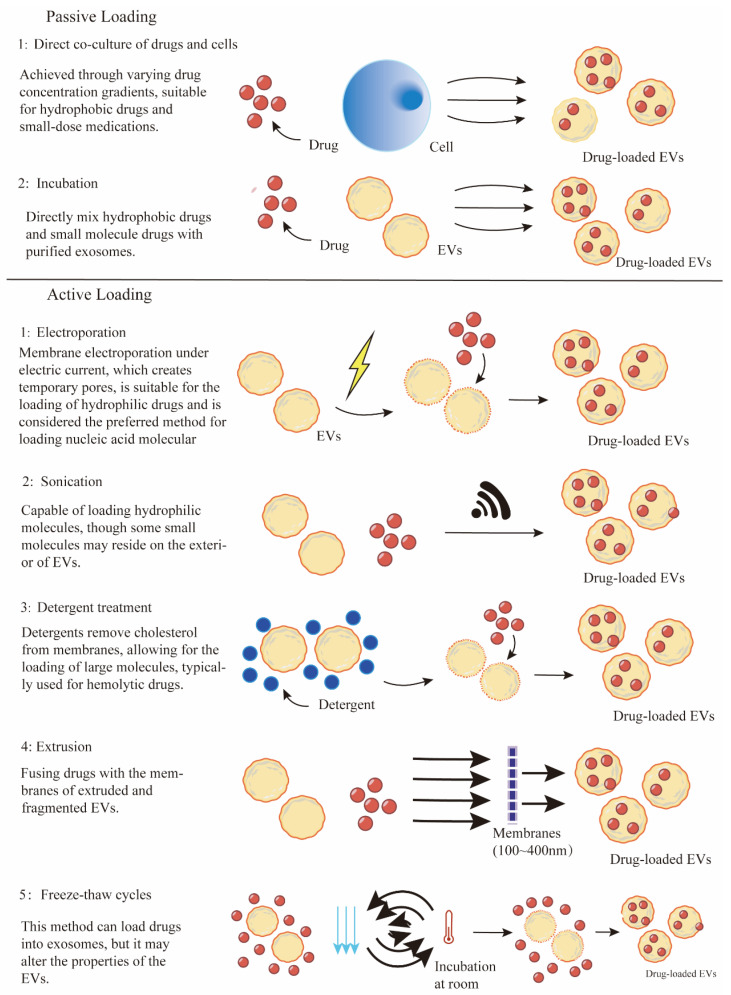
Various technologies for drug loading into EVs.

EVs are promising carriers for a variety of therapeutic agents, including chemotherapeutic drugs, nucleic acids, and proteins. The efficiency of drug loading into EVs is a fundamental aspect of their utility as drug delivery vehicles (Figure 2). In general, strategies utilized for cargo being loaded into EVs can be simply divided into two categories: (1) Pre-loading methods—drug loading before isolation of EVs and (2) Post-loading methods—drug loading after isolation of EVs. The examples of EVs loaded with therapeutics through different strategies for anti-cancer drug delivery are summarized in Table 1.

#### 3.3.1. Pre-Loading Methods

Pre-loading methods are endogenous cargo-loading strategies that involve incorporating drugs into EVs through biogenesis processes. These approaches handle the cells as EV manufacturing equipment, requiring the addition of various raw materials to produce customized EVs. Thus, effectively loading various types of cargo molecules into the parent cells is one of the important processes.

For small molecule chemotherapeutics, co-incubation is considered a practical strategy to facilitate their entry into parent cells, which then produce drug-loaded EVs. During this process, drugs are added to the culture medium, allowing them to cross the lipid bilayer, enter the cells, and subsequently be sorted into EVs via endogenous biogenesis machinery. This strategy offers the advantages of convenience and maintains the structure of EVs, whereas the loading efficiency is significantly affected by drug characteristics and incubation conditions [146]. Hydrophobic molecules are often used as typical drugs for EV loading due to their easy interaction with lipids on the EV surface. For example, direct incubation was used as an approach for applying EVs as hydrophobic photosensitizer delivery systems [147]. Photosensitizers, including Phthalocyanine chloride tetrasulfonic acid (AlPcS4) and AIE-photosensitizer MBPN-TCyP, were loaded into EVs by incubation, thereby enhancing the photodynamic therapy (PDT) effect [148,149]. Additionally, chemotherapeutic drugs, including paclitaxel (PTX), doxorubicin (DOX), hydroxyl camptothecin, cisplatin, and methotrexate (MTX) [80,150,151], have been investigated for loading into EVs by the co-incubation method. Recent studies indicated that proper stimuli such as ultraviolet or low-current electricity could enhance EV drug loading efficiency and secretion [150,152,153]. Therefore, to improve the drug loading efficiency, it is crucial to customize the co-incubation conditions, including drug concentrations, incubation times, parent cell types, and appropriate stimulus.

For therapeutic nucleic acids or proteins, transfection of specific plasmids into parent cells using selected chemical reagents or viruses stands as the most commonly used approach to induce transient or stable overexpression. These overexpressed cargos are incorporated into EVs by utilizing the endogenous biogenesis processes. For example, hepatocyte growth factor (HGF) siRNA was loaded into EVs after transfection of HEK293T cells, resulting in suppression of tumor growth and angiogenesis in gastric cancer [154]. Chimeric LAMP2b-DARPin G3 gene were transduced into HEK293T with the aim to label DARPin G3 on the surface of EVs, facilitating their specific binding to HER2/Neu and delivering siRNA molecules [155]. Similarly, the fusion expression of the exosome-enriched membrane protein prostaglandin F2 receptor negative regulator (PTGFRN) with IL12 facilitated the surface display of IL12 exosomes (exoIL12), inducing significantly improved antitumor immunity ability [156]. These endogenous methods offer the advantages of high repeatability and simplicity, and they can be designed to improve EV loading efficiency and targeting ability by altering the expression profile of the cells. However, some disadvantages, such as limited transfection efficiency, genetic instability, time consumption, high dependence on cell viability, and difficulty controlling the number of drugs loaded into EVs, need to be addressed for their extensive clinical application.

#### 3.3.2. Post-Loading Methods

Post-loading methods are exogenous cargo loading strategies that involve the direct loading of exogenous cargo into EVs after the isolation of EVs. These strategies are mainly divided into passive loading and active loading methods. The commonly used post-loading approaches consist of direct incubation, transfection, electroporation, sonication, extrusion, and freeze-thaw cycles [157].

For several hydrophobic or some small-molecule drugs that can passively diffuse across the lipid bilayer, simple incubation with these drugs provides possibilities for their directly integrating into the isolated EV lipid bilayer. Similar to the co-incubation method in pre-loading, various hydrophobic drugs such as paclitaxel (PTX), doxorubicin (DOX), curcumin, chlorin e6 (Ce6), Zinc Phthalocyanine have been directly encapsulated into purified EVs by mixing at designed conditions [126,158,159,160,161,162,163,164,165]. This passive incubation loading method is feasible and easy to operate without requiring special equipment. Incubation exhibits protection for cargo properties and membrane integrity of EVs, whereas the loading efficiency is associated with the nature of drugs [126]. The limitations of passive loading application are the relatively low loading efficiency and the hydrophobic lipid membrane, which presents a major obstacle for loading hydrophilic drugs into the aqueous EV.

For hydrophilic therapeutics that have difficulty penetrating the lipid membrane of EVs, various encapsulation mechanisms have been developed. These methods, known as active loading strategies, aim to bypass the EV membrane through chemical or physical induction.

*Chemical induction methods* Chemical induction strategies utilize chemical agents such as transfection reagents and membrane permeabilizers to facilitate drug entry into EVs. Transfection techniques, including the use of employing agents such as liposomes and calcium chloride, are well-established for improving the loading of hydrophilic drugs into EVs. These agents are frequently used to facilitate the internalization of nucleic acids by interacting with the lipid membrane [166,167,168]. A notable method that combines calcium chloride transfection with heat shock was developed to effectively load small RNAs into EVs [169]. Furthermore, the modification of siRNA/miRNA with hydrophobic cholesterol significantly enhances their loading efficiency into EVs through passive insertion [163,170,171,172]. Another approach involves the use of surfactant compounds or detergents to chemically permeabilize the EV membrane, allowing for enhanced molecular passage. Saponin, a plant-derived surfactant, has proven effective in creating membrane pores by extracting cholesterol and facilitating the entry of various molecules [173]. This method was successfully used to increase the loading capacity of catalase, an antioxidant enzyme crucial for protecting neuronal tissues from oxidative stress [174]. Additionally, saponin treatment also improved the encapsulation efficiency for small hydrophilic molecules compared to passive loading methods [175] and was reported to facilitate the encapsulation of hollow gold nanoparticles [176]. However, due to its potential to cause hemolysis [177], the concentration of saponin should be carefully controlled, and thorough purification of EVs is necessary to eliminate any residual saponin.

*Physical induction methods* Physical induction strategies enhance the permeability of EV membranes through the application of external forces. These methods mainly include electroporation, sonication, freeze-thaw cycles, and extrusion. Each technique transiently alters the EV membrane to facilitate the incorporation of therapeutic agents.

Electroporation is the most commonly used technique for incorporating nucleic acids and therapeutic agents into EVs. This method involves mixing purified EVs with therapeutic cargo in an electroporation buffer, followed by the application of an electric field to generate transient pores in the EV membranes, facilitating cargo incorporation [178,179]. Electroporation has been effectively utilized to load a variety of cargos, particularly siRNA or miRNA, which generally do not diffuse spontaneously into EVs due to their relatively large size [180,181]. For example, electroporation was applied for the loading of antisense oligonucleotides (ASO), Cas9 mRNA, and guide RNAs into RBC-derived EVs, significantly enhancing miRNA inhibition and CRISPR-Cas9 genome editing capabilities [122]. This technique was also employed to encapsulate chemotherapeutic agents, such as doxorubicin (DOX), into iRGD peptide-labeled EVs, markedly improving targeting and delivery to breast cancer cells overexpressing integrins [99]. Similarly, doxorubicin (DOX) was successfully loaded into EVs derived from lens epithelial cells via electroporation, demonstrating effective uptake and substantial anti-proliferative effects [182]. An optimized electroporation protocol for drug loading of EVs resulted in a 190-fold increase in drug efficacy compared to free doxorubicin (DOX) [179]. Moreover, 5-fluorouracil (5-FU) and a miR-21 inhibitor were co-loaded into EVs via electroporation for targeted delivery to colon cancer cells to mitigate drug resistance [183]. Despite its advantages, electroporation may induce EV aggregation and structural instability, potentially reducing the effectiveness of the delivery system [184]. Thus, optimizing electroporation parameters, including the drug-to-EV ratio, buffer composition, pulse capacitance, and electric field strength, is crucial to preserve the functionality of the drug delivery vehicles.

Sonication is another promising physical method for loading drugs into EVs, which utilizes ultrasound energy to facilitate transient membrane deformation, significantly enhancing the diffusion of drugs into EVs. Sonication is suitable for loading both hydrophobic and lipophilic cargos into EVs during co-incubation. For example, siRNA was loaded into EVs by sonication to downregulate the oncogenic receptor tyrosine kinase Her2 [185]. Moreover, anticancer agents such as doxorubicin (DOX), paclitaxel (PTX), erastin (a ferroptosis inducer), rose bengal (a photosensitizer), and curcumin were also incorporated into EVs through sonication to improve therapeutic outcomes [97,186,187,188]. However, sonication has relatively low loading efficiency for hydrophobic drugs and may cause EV aggregation [189]. Liu et al. demonstrated that integrating traditional sonication with microfluidic technologies can enhance cargo loading efficiency [190]. Consequently, sonication is also a promising technique used in combination with other drug-loading methods to optimize the EV cargo loading capacity.

Extrusion, a method originally developed for synthesizing liposomes, involves repeatedly forcing a mixture of EVs and therapeutic agents through membranes with nanoscale pores [191]. This process disrupts the EV membranes, facilitating vigorous mixing with the drugs and enabling the incorporation of exogenous compounds into the EVs [192]. For example, paclitaxel (PTX) was loaded into EVs from various sources via extrusion, demonstrating effectiveness in cancer therapy [193]. Extrusion was also employed to load doxorubicin (DOX) into EVs, significantly reducing the viability of cancer cells [194,195]. Additionally, protein catalase was also incorporated into EVs through serial extrusion, targeting inflammatory and neurodegenerative disorders [192]. Recent comparative studies indicate that extrusion offers superior packaging efficiency relative to methods such as incubation and electroporation [196]. However, repeated extrusion can alter the structural integrity of EVs, including changes in size, zeta potential, and composition, which may lead to decreased delivery efficacy and potential adverse reactions [197].

Freeze and thaw cycles leverage thermal energy to facilitate drug loading into EVs. This technique involves subjecting EVs to repeated cycles of freezing and thawing, which induces the formation of ice crystals. These crystals temporarily disrupt the EV membrane, allowing hydrophilic compounds to penetrate the EV interior. The membrane reconstitutes when the ice melts during thawing [198]. Typically, drugs are incubated with EVs at room temperature for a designated period before being rapidly frozen at −80 °C or in liquid nitrogen and then thawed at room temperature. This cycle is repeated at least three times to ensure effective drug encapsulation [199]. While the freeze-thaw technique has been employed to load several therapeutic cargos into EVs [174,200,201,202], it generally exhibits lower protein loading efficiency compared to sonication and extrusion methods [189,197,203]. Additionally, increasing the number of freeze-thaw cycles can induce EV aggregation and alter membrane protein levels, resulting in decreased EV concentration and increased size [204]. Therefore, it is crucial to optimize the freeze-thaw conditions, including temperature and cycle duration, to maximize drug loading efficiency in EVs.

In addition to the previously described strategies, several other approaches are applied to enhance therapeutic loading into EVs. Hypotonic dialysis, for example, demonstrated superior drug loading efficiency with porphyrins compared to direct incubation, electroporation, and extrusion [175]. Hybridization techniques, such as fusing EVs with liposomes, were developed to improve drug loading capacity, tumor homing, and circulation properties [202,205]. A promising set of strategies involves engineering parent cells to enrich specific biomolecules within EVs. For example, proteins can be incorporated into EVs through fusion with endogenously EV-enriched proteins like CD63, CD9, and LAMP2B. Notably, the ovalbumin (OVA) antigen was fused expression with CD63 to prepare the OVA-loaded EVs, significantly improving the immunogenicity of DNA vaccines and inhibiting tumor growth [206]. Yim et al. introduced a light-inducible loading system that integrates a reversible protein-protein interaction module (photoreceptor cryptochrome 2, CRY2, and CIB-interacting basic-helix-loop-helix 1, CIB1) with exosome biogenesis for delivery of interesting proteins. In this system, a truncated version of CIB1 (CIBN) was fused with the exosome-enriched protein CD9, while CRY2 was conjugated with cargo proteins. Upon blue light illumination, CRY2 and CIBN bind reversibly, facilitating efficient loading or release of cargo proteins [207]. Recently, Bui et al. developed a drug-inducible cargo loading system using the FRB/FKBP heterodimerization system, in which FKBP (FK506 binding protein) and FRB (FKBP rapamycin binding) domains dimerize when there is a biologically innocuous rapamycin analog drug. Here, the EV membrane protein CD63 was conjugated with FKBP2, and FRB was fused with the cargo proteins of interest. Induction by a rapamycin analog triggers FKBP-CD63 to bind FRB-tagged cargo proteins, enhancing their loading into EVs [208]. These bioengineering strategies are not only used to improve loading efficiencies but also applied to enhance the tumor-targeting capabilities of EVs, which is one of the important aspects of the following discussion.

**Table 1:** Examples of EVs loaded with therapeutics for cancer treatment.

Cargo Loading Method	Therapeutic Cargo	Sources of EVs	Cancer Type	Function	Study Type	Year	Ref.
Pre-loding, coincubation	Phthalocyanine chloride tetrasulfonic acid (AlPcS4)	Gastric cancer MGC803 cells	Gastric cancer	Deconstruct exosome for releasing Dox and enable the photodynamics for combination therapy	In vitro and in vivo	2021	[148]
Pre-loding, coincubation	AIE-photosensitizer MBPN-TCyP	Dendritic cells	Breast cancer and colorectal cancer	Synergistic photodynamic immunotherapy elicits dramatic anti-tumor immune responses	In vitro and in vivo	2022	[149]
Pre-loding, coincuba-tion	MTX	Mouse hepatocarcinoma tumour cells H22	Hepatocarcinoma	Inhibit ascites hepatocarcinoma growth without typical side effects	In vitro and in vivo	2012	[80]
Pre-loding, coincubation	Cisplatin/PTX	Human ovarian cancer tumour cells A2780	Ovarian cancer	Inhibit human ovarian cancer growth without affecting liver and kidney functions of SCID mice	In vitro and in vivo	2012	[80]
Pre-loding, coincubation	MTX	Mouse fibroblast cells L929	Glioblastoma	Facilitate extravasation across BBB and inhibit human brain tumor growth	In vitro and in vivo	2018	[150]
Pre-loding, coincubation	MTX	Primary malignant cells that are frequently accompanied by malignant pleural effusion (MPE) in their advanced stages	Lung cancer and colon cancer with MPE	Exhibit clinical activity in killing tumor cells and TAMs and induce antitumor immune responses	In vitro and in vivo	2019	[152]
Pre-loding, coincubation	ICG and PTX	HEK293T	Breast cancer	Increase the anticancer activity through combination of chemo/photothermal/photodynamic therapy	In vitro and in vivo	2022	[151]
Pre-loding, coincubation	PTX	BM-MSCs (SR4987)	Pancreatic adenocarcinoma	Exhibit strong antiproliferative activity on human pancreatic adenocarcinoma cells CFPAC-1	In vitro	2014	[78]
Pre-loding, transfection	HGF siRNA	HEK293T	Gastric cancer	Suppress proliferation and migration of both cancer cells and vascular cells	In vitro and in vivo	2018	[154]
Pre-loding, electroporat	PTX/miR-16/Penicillin/MCP-1/Cas9-GFP	Differentiated human promyelocytic leukemia cells (dHL-60) and naïve HL-60	Breast cancer cells (MCF-7)/Colon cancer cells (COLO205)/Jurkat cells	Dhl60 exhibit increased drug loading and production efficiency	In vitro	2012	[209]
Pre-loding, transduction and coincubation	TRAIL and Cabazitaxel (CTX)	MSCs	Oral squamous cell carcinoma	Synergistically inhibit the growth of cancer cells by inhibiting the activation of PI3K/Akt/mTOR pathway and inducing apoptosis	In vitro and in vivo	2020	[210]
Pre-loding, transduction	miR-379	MSCs	Breast cancer	Inhibit the growth of breast cancer by downregulating cyclooxygenase (COX-2)	In vitro and in vivo	2017	[211]
Post-loding, coincubation	DOX	Brain endothelial cells	Brain cancer	Mediate drug delivery across the BBB and exert cytotoxic efficacy against brain cancer in zebrafish	In vitro and in vivo	2015	[158]
Post-loding, coincubation	Curcumin	Bovine milk	Multiple cancers (breast, lung and cervical cancer)	Enhance antiproliferative activity against multiple cancer cell lines (breast, lung, and cervical cancer) and e cervical tumor xenograft	In vitro and in vivo	2017	[159]
Post-loding, coincubation	Withaferin A (WFA)/Bilberry-derived anthocyanidins (Anthos)/Curcumin (Cur)/paclitaxel (PTX) and docetaxel (DOC)	Bovine milk	Lung cancer and breast cancer cells	Enhance anti-cancer and anti-inflammatory effects	In vitro	2016	[126]
Post-loding, coincubation for Ce6/electroporation for siRNA	Ce6/PD-L1 siRNA	NK cells	Hepatocellular carcinoma and Colon cancer	Effectively inhibit cancer progression by effective PDT or restoring the immunological surveillance function	In vitro and in vivo	2022	[160]
Post-loding, coincubation	Zinc Phthalocyanine	Metastatic murine melanoma cells (B16F10)	Colon cancer	Increase efficacy and selectivity of PDT	In vitro and in vivo	2021	[161]
Post-loading, coincubation	Zinc Phthalocyanine	M1/M2-like macrophages/B16F10/Milk	Colon cancer	Increase photodynamic therapy and promote immunological memory	In vitro and in vivo	2022	[162]
Post-loading, coincubation	DOX/Cholesterol-modified miRNA 159	Human monocytes (THP-1)	Triple negative breast cancer (TNBC)	Co-delivering miR159 and Dox by targeted Exo for TNBC therapy	In vitro and in vivo	2019	[163]
Post-loading, coincubation	Cholesterol-modified miRNA 34a	HEK293T	Oral squamous cell carcinoma	Inhibition of oral squamous carcinoma HN6 cell proliferation, migration, and invasion by down regulating SATB2 expression	In vitro	2022	[172]
Post-loading, coincubation	DOX	RAW 264.7 cells pre-treated with hyaluronic acid (HA) and the β-blocker carvedilol (CV)	Breast cancer	Enhance the antitumor effects of DOX	In vitro and in vivo	2022	[164]
Post-loading, coincubation	DOX/Chemosensitizer lonidamine (LND)	Non-small cell lung carcinoma A549 cells	Non-small cell lung carcinoma	Synergistically increase anticancer activity	In vitro and in vivo	2022	[165]
Post-loading, calcium chloride transfection combined with heat shock/electroporation	miR-15a mimic/inhibitor	THP-1 cells	NA	Effectively enhance miRNA loading efficiency to EVs	In vitro	2017	[169]
Post-loading, transfection	miR-335	Human hepatic stellate cell LX2	Hepatocellular carcinoma	Inhibit hepatocellular carcinoma growth	In vitro and in vivo	2018	[167]
Post-loading, transfection	VEGF siRNA	Brain endothelial bEND.3 cells	Brain cancer	Mediate siRNA Delivery across the BBB to inhibit brain tumor growth	In vitro and in vivo	2017	[168]
Post-loading, saponin	DOX	Human GBM cell line U87 and U251 cells	Glioblastoma	Eliminate the original cargos of glioblastoma cell-derived small EVs for efficient drug delivery	In vitro and in vivo	2022	[212]
Post-loading, saponin/electroporation/extrusion/dialysis	Porphyrins	HMSCs/HUVECs/MDA-MB-231 cells	Breast cancer MDA-MB-231 cells	Induce a stronger phototoxic effect than free drug in a cancer cell model	In vitro	2015	[175]
Post-loading, saponin/sonication/extrusion/freeze-thaw cycles	Catalase	Raw 264.7	Neuronal cells PC12	Exhibit high loading efficiency, sustained release, and catalase preservation against proteases degradation and provide significant neuroprotective effects	In vitro and in vivo	2015	[174]
Post-loding, electroporation	DOX	HEK293F/B16F10	Metastatic murine melanoma B16F10 cells	Optimized electroporation improves the loading of EVs with DOX	In vitro	2022	[179]
Post-loding, electroporation	ASOs/Cas9 mRNA and gRNA	Red blood cells (RBCs)	Leukemia/breast cancer	Exhibit highly robust microRNA inhibition and CRISPR–Cas9 genome editing	In vitro and in vivo	2018	[122]
Post-loding, electroporation for siRNA; Pre-loading, co-incubation for DOX	KRAS^G12D^ siRNA/DOX	Human umbilical cord mesenchymal stromal cells (UC-MSCs)	Pancreatic ductal adenocarcinoma (PDAC)	Co-delivery KRAS^G12D^ siRNA and DOX to PDAC cells to inhibit the cancer progression	In vitro	2023	[181]
Post-loding, electroporation	ITGB6 siRNAs	Prostate cancer cells (DU145 and PC3)	Prostate cancer	Delivery of siRNAs targeting the ITGB6 to inhibit adhesion and migration of recipient prostate cancer cells	In vitro	2022	[180]
Post-loding, sonication	HER2 siRNA	HEK293T/MCF-7	Breast cancer	Knockdown of HER2, a therapeutic target that is overexpressed in numerous cancers	In vitro	2016	[185]
Post-loding, soni-cation	DOX	RAW 264.7	TNBC	Significantly inhibit TNBC tumor growth	In vitro and in vivo	2020	[97]
Post-loding, sonication	PTX	RAW 264.7 macrophages	Lung cancer	Inhibit growth of pulmonary metastases and overcome MDR in Cancer cell	In vitro and in vivo	2016	[186]
Post-loding, sonication	Erastin/Rose Bengal	HEK293T	Hepatocellular carcinoma	Induce obvious ferroptosis in HCC with minimized toxicity in liver and kidney	In vitro and in vivo	2021	[187]
Post-loding, extrusion	PTX	Mesenchymal stem cells (MSCs)	Breast cancer	Exhibit therapeutically efficient for the treatment of breast cancer	In vitro and in vivo	2018	[193]
Post-loding, extrusion for DOX/electroporation for P-gp siRNA	DOX/P-gp siRNA	Normal ovarian epithelial Iose80 cells	Ovary cancer	Target delivery of chemotherapeutics to overcome drug resistance of ovarian cancer	In vitro and in vivo	2023	[194]
Post-loding, extrusion	DOX	HT1080/Hela	Fibrosarcoma	Tumor cell-derived exosomes preferentially targeted their cell of origin	In vitro and in vivo	2020	[195]
Post-loding, freeze-thaw cycles	Liposome	Mouse fibroblast sarcoma-derived CMS7-wt/CMS7-HE (HER2 overexpression)/Raw 264.7	HeLa cells	Develop hybrid exosomes by fusing the membranes of exosomes with liposomes for loading therapeutic agents into exosomes	In vitro	2016	[199]
Post-loding, freeze-thaw cycles/extrusion,/sonication	DOX	Platelets	Breast cancer	Efficiently load DOX and kill breast cancer cells	In vitro	2023	[201]
Post-loding, freeze-thaw cycles	Folate-modified Liposomes with or without PTX	Mesenchymal stem cells (MSCs)	Colon carcinoma cell line CT26/Mouse melanoma cell line B16/Human ovarian cancer cell line A2780	Increase therapeutic potential of PTX for cancer therapy	In vitro and in vivo	2024	[202]
Fused expression with tetraspanin CD63	OVA	293F cells	Immune cells	Significantly inhibit tumor growth by induce DNA vaccine-specific CD8^+^ T cell responses	In vitro and in vivo	2017	[206]
Fused expression with CD9 (CIBN and CRY interaction system)	Proteins: mCherry/luciferase/Bax/super repressor IκB (srIκB)/Cre recombinase	HEK293T	HeLa cells/Rat embryonic primary neurons/Neuronal cells	Significantly increase intracellular levels of cargo proteins and their function in recipient cells	In vitro and in vivo	2016	[207]
Fused expression with CD63 (FRB/FKBP heterodimerization system)	Proteins: Diphtheria toxin A (DTA)	DTA-resistant HT1080 cells	HT1080 cells	Efficient system enables to load any protein-based therapeutics into EVs	In vitro	2023	[208]

## 4. Extracellular Vesicles Modification for Targeted Anti-Cancer Drug Delivery

EVs have gained attention as promising carriers for anti-tumor therapeutics, owing to their unique properties and cargo-carrying capabilities. However, their clinical application is limited by significant accumulation in the liver and spleen, where they are rapidly cleared by macrophages [41], highlighting the necessity for advanced targeting strategies to enhance the precision of therapeutic delivery. To optimize therapeutic cargo delivery, substantial efforts are now focused on improving the organ-specific and molecular targeting of EVs through various EV modification strategies. In the following sections, we explored modification strategies that involve the incorporation of exogenous surface ligands to significantly enhance the targeting efficacy and therapeutic potential of EVs.

### 4.1. Genetic Target Engineering

Genetic engineering represents a convenient and extensively researched approach for modifying donor cells to impart EVs with new properties. In this strategy, donor cells are transfected with the plasmids that encode fusion genes, combining targeting ligands with transmembrane proteins located on the EV surface. Consequently, these transfected cells secrete engineered EVs that display the targeting ligands, enhancing their specificity for particular tissues or cells. Examples of genetically engineered EVs for targeted anti-cancer drug delivery are summarized in Table 2.

#### 4.1.1. Genetic Target Engineering by Fusion Expression with LAMP-2B

LAMP-2B (lysosomal-associated membrane glycoprotein 2b) is the most widely used exosome transmembrane protein to display targeting motifs. It is demonstrated that the N-terminus of LAMP-2B, which is exposed on the exosome surface, can be modified to incorporate targeting sequences [15,213]. Structurally, human LAMP-2B consists of a 29 amino acid signal peptide, a large N-terminal extramembrane domain, and a C-terminal transmembrane region followed by a very short cytoplasmic tail [214]. To enhance the specificity and efficiency of EV delivery, various targeting ligands such as cell-specific binding peptides, antibody fragments, or targeting proteins have been successfully fused to this extracellular domain at the N-terminus of LAMP-2B.

*iRGD peptide (amino acid sequence: CRGDKGPDC)* The iRGD peptide enhances the extravasation and specific penetration into tumors due to its high affinity for integrins (αvβ3, αvβ5) and neuropilin-1 (NRP-1) on tumor vascular endothelium and tumor cells, showing significant potential for tumor targeting [215,216]. Targeted delivery of EVs to tumor cells can be facilitated by fusing the EV membrane protein, Lamp2b, with the integrin-specific peptide, iRGD. For example, Zhao et al. prepared iRGD-modified exosomes by transfecting HEK293T cells with iRGD peptide-Lamp2b plasmids. The isolated iRGD-exosomes were subsequently loaded with miR-484 by electroporation, thereby inhibiting angiogenesis and enhancing chemotherapy sensitivity in ovarian cancer and endothelial cells [217]. Similarly, Tian et al. produced iRGD-modified exosomes by genetically modifying immature mouse dendritic cells to express the iRGD peptide and Lamp2b fusion plasmids. The resultant iRGD-exosomes, loaded with doxorubicin (DOX) via electroporation, demonstrated efficient targeting and drug delivery ability to αvβ3 integrin-positive breast cancer, significantly reducing tumor growth without noticeable toxicity [99]. Further, iRGD-modified EVs loaded with doxorubicin (DOX) were also investigated to enhance internalization by glioblastoma cells, improving drug delivery across the blood-brain barrier (BBB) [218]. Additionally, Zhou et al. utilized iRGD-exosomes as a delivery system for KRAS siRNA targeting integrin αvβ3-bearing lung cancer cells, achieving specific KRAS gene silencing and tumor suppression [219]. In the realm of metabolic targeting, iRGD-exosome-mediated delivery of siRNA of carnitine palmitoyl transferase 1A (CPT1A), a key enzyme in fatty acid oxidation (FAO), was shown to effectively reverse oxaliplatin resistance and inhibit tumor growth in colon cancer by disrupting the FAO metabolic pathways, with a high safety profile in vivo [220]. In 2022, iRGD-modified exosomes were employed to deliver BCL2 siRNA to diffuse large B-cell lymphoma cells, resulting in tumor growth inhibition without significant toxicity [221].

*tLyP-1 peptide (amino acid sequence: CGNKRTR)* The tLyp-1 peptide is a heptapeptide that selectively targets neuropilin-1 (NRP1) and neuropilin-2 (NRP2), receptors highly expressed in various tumor tissues, including non-small cell lung cancer (NSCLC), playing critical roles in cancer drug delivery systems [222]. tLyp-1 acts as a tumor-homing peptide that not only targets tumors but also penetrates tumor blood vessels and stroma, reaching deep into the tumor parenchyma [222,223]. Therefore, tLyp-1-modified exosomes were designed for active targeting and extensive penetration into the tumor parenchyma. In 2020, tLyp-1-modified exosomes were prepared by transfection of HEK293T cells with tLyp-1-Lamp2b fusion plasmid. These exosomes were subsequently loaded with SOX2 siRNA through electroporation, effectively silencing SOX2 expression in lung cancer cells and diminishing the stemness of cancer stem cells [223,224].

*Fragment of Interleukin 3* The interleukin-3 (IL-3) fragment, a native ligand for the interleukin-3 receptor α, is overexpressed on chronic myelogenous leukemia (CML) blasts relative to normal hematopoietic cells, making it a viable target for cancer drug delivery systems [225,226]. To exploit this targeting capability, exosomes were engineered by fusing the IL-3 fragment with Lamp2b in 293T cells. These modified exosomes, designated as IL3-exosomes, were subsequently loaded with Imatinib or BCR-ABL siRNA via direct incubation or transfection techniques. The resulting formulations, IL3-Exo-Imatinib and IL3-Exo-BCR-ABL siRNA demonstrated specific targeting ability against CML cells and effectively inhibited cancer cell growth both in vitro and in vivo [227].

*HER2-binding affibody Z_HER2_ (amino acid sequence: VDNKFNKEMRNAYWEIALLPNLNNQQKRAFIRSLYDDPSQSANLLAEAKKLNDAQAPK)* Affibody molecules, a class of high-affinity proteins identified through phage display technology, exhibit specific binding properties suitable for targeting desired molecules. Due to their small (~6 kDa) and robust structure, affibody molecules are particularly effective for tumor targeting in diagnostic and therapeutic applications [228]. In 2004, Wikman et al. first developed a Her2-specific binding affibody demonstrating nanomolar affinity [229]. Subsequently, in 2020, the HER2-binding affibody Z_HER2_ was fused to the N-terminus of LAMP-2 to product Z_HER2_-Exosome (Z_HER2_-Exo). After being loaded with 5-fluorouracil (5-FU) and miRNA-21 inhibitor via electroporation, this Z_HER2_ tagging exosomes enabled specifically co-delivery of these agents to HER2-expressing colon tumors, significantly enhancing cytotoxicity against 5-FU-resistant colon cancer cells [183].

*HER2-specific DARPin G3* Designed ankyrin repeat proteins (DARPins) are a class of synthetic peptides selected for their specificity in recognizing a broad range of target proteins. DARPins are characterized by their lack of disulfide bonds and high affinity for their ligands [230]. In 2007, Plueckthun et al. developed a HER2-specific DARPin, G3, with a picomolar affinity for HER2, which is overexpressed in various tumors, including breast, ovarian, and gastric cancers [231]. In 2019, HER2-specific DARPin-modified exosomes were purified from HEK293T cells stably expressing DARP-LAMP2, enabling targeted therapeutic applications. These HER2 DARPin-Exo were subsequently loaded with TPD52 siRNA via electroporation, achieving up to 70% downregulation of TPD52 expression in HER2-positive breast cancer cells [155]. Similarly, bone marrow mesenchymal stem cells (BM-MSCs) were utilized to produce HER2-specific DARPin-modified exosomes, which were then loaded with doxorubicin (DOX) via electroporation. This approach facilitated specific delivery of doxorubicin (DOX) to HER2-positive breast cancer cells, significantly inhibiting tumor growth [232]. Additionally, DARPins G3-modified exosomes were radiolabeled with 99mTc, creating 99mTc-exosomes that accumulated in HER2-positive SKOV-3 xenograft tumor tissue, enabling tumor visualization via planar imaging [233].

#### 4.1.2. Genetic Target Engineering by Fusion Expression with PDGFR TM Domain

PDGFR TM (transmembrane domain of platelet-derived growth factor receptor) is often used as an effective fusion partner to display targeting ligands on exosome surfaces. This single-chain transmembrane glycoprotein facilitates the presentation of various targeting agents, including polypeptides and antibodies, enhancing the specificity and efficacy of exosome-mediated delivery systems [234,235].

*GE11 peptide (amino acid sequence: YHWYGYTPQNVI)* The GE11 peptide, identified through phage display peptide library screening, specifically binds to the epidermal growth factor receptor (EGFR), which is overexpressed in various human cancers [236]. In 2013, GE11-modified exosomes were engineered in HEK293T cells by fusing GE11 with the PDGFR transmembrane region. Subsequently, let-7a miRNA was loaded into these exosomes via transfection. The resulting GE11-Exo-let-7a miRNA demonstrated a high affinity for EGFR-overexpressing cancer cells and significantly reduced tumor growth, highlighting the potential of GE11-modified exosomes as an effective delivery system for targeted EGFR therapy [237].

*Single-chain variable fragment (scFv) antibodies against CD3 and EGFR* The single-chain fragment variable (scFv), which contains the complete antigen-binding domains of a whole antibody, possesses numerous advantages, including high specificity and affinity for antigens, low immunogenicity, and the ability to penetrate and diffuse through tumor tissues [238]. scFv has been used as highly effective and specific targeting motifs for EVs [239]. In 2018, Cheng et al. developed an αCD3/αEGFR synthetic bivalent antibodies by genetically linking scFv antibodies targeting human T cell CD3 (UCHT1) and human EGFR (cetuximab). This αCD3/αEGFR was further displayed on the exosomal surface in Expi293F cells by fusion with the human PDGFR TM domain, developing synthetic multivalent antibodies retargeted exosomes (SMART-Exos). Additionally, αEGFR and αCD3 scFv-modified exosomes were separately produced as controls, using the PDGFR TM domain for fusion. The resulting SMART-Exos, targeting both T-cell surface CD3 and EGFR-expressing triple-negative breast cancer (TNBC) cells, demonstrated significant antitumor efficacy both in vitro and in vivo [240].

#### 4.1.3. Genetic Target Engineering by Fusion Expression with Lactadherin C1–C2 Domain

Lactadherin is a membrane-associated protein that is highly enriched in the outer exosome membrane due to its interaction with phosphatidylserine [241]. Studies have shown that targeting ligands can be efficiently fused to the C1–C2 domain of lactadherin to improve the targeted delivery capabilities of EVs.

*Single-chain variable fragment (scFv) antibodies against HER2* The anti-HER2 scFv ML39 was genetically fused to the lactadherin C1-C2 domain in HEK293 cells, producing ML39-modified exosomes. These were subsequently loaded with HChrR6 mRNA by electroporation, developing the ML39-Exo-HChrR6 mRNA delivery system, referred to as EXO-DEPTs. HChrR6, an optimized bacterial enzyme, converts CNOB (6-chloro-9-nitro-5-oxo-5H-benzo-(a)-phenoxazine) into the strong fluorescent and cytotoxic drug MCHB (9-p-amino-6-chloro-5H-benzo[a]phenoxazine-5-one), enabling specific targeting and significant cytotoxicity against HER2-expressing cells and markedly inhibiting the growth of orthotopic HER2 breast cancer tumors [242,243]. Longatti et al. (2018) used three different scFvs with varying affinities (high, intermediate, and low) for HER2 to modify the exosomes. These anti-HER2 scFvs exosomes were further labeled with CFSE (5(6)-Carboxyfluorescein diacetate N-succinimidyl ester) and applied to monitor the targeting delivery ability to cancer cells with different HER2 expression levels, demonstrating that both high-affinity scFv and high receptor expression were parameters positively influencing the selective uptake [239]. In another study, two copies of a HER2 polypeptide ligand were fused to the C-terminal C1-C2 domains using a lentivector in HEK293 cells pre-engineered to stably express HER2 miRNA. The resulting exosomes, named 293-miR-XS-HER2, showed increased specificity and enhanced anti-tumor efficacy in targeted drug delivery [244].

#### 4.1.4. Genetic Target Engineering by Fusion Expression with the Tetraspanin Superfamily Proteins

The tetraspanins superfamily proteins are involved in organizing membrane microdomains, specifically tetraspanin-enriched microdomains (TEMs), by clustering and interacting with a wild variety of transmembrane and cytosolic signaling proteins [245]. Tetraspanins, including CD9, CD37, CD81, CD63, and CD82, are predominantly enriched in the membranes of exosomes, serving as key biomarkers for these vesicles [246]. The tetraspanins feature two extracellular loops, which can be utilized to display targeting sequences or probes. For example, the fluorescent protein pHluorin was inserted into the small extracellular loop to monitor exosome secretion and uptake [247]. Apo-A1, the principal component of high-density lipoprotein (HDL), targets the scavenger receptor class B type 1 (SR-B1), a receptor known for mediating phospholipid transfer between HDL and the cell membrane [248]. Notably, SR-B1 is overexpressed on the surface of various liver cancer cells, including HepG2, hepatic carcinoma, melanoma, and prostate cancer [249,250,251]. In 2018, ApoA-1 was genetically inserted into the small extracellular loop of CD63, enabling its presentation on the surface of exosomes as a fusion protein. These engineered Apo-A1 exosomes, produced by 293T cells and loaded with miR-26a through electroporation, demonstrated selective internalization by HepG2 cells via SR-B1 receptor-mediated endocytosis, significantly inhibiting cell proliferation and migration [252].

#### 4.1.5. Genetic Target Engineering by Fusion Expression with the CD47

CD47 is a transmembrane protein abundant on the surface of exosomes. Yang et al. modified the surface of exosomes by fusing CDX peptide (amino acid sequence: FKESWREARGTRIERG) targeting PTEN-deficient human U87 glioblastoma cells and CREKA peptide targeting PTEN-deficient mouse GL261 glioblastoma cells to the N-terminal of CD47 on the external exosomal surface. These CDX/CREKA-modified exosomes were subsequently loaded with PTEN mRNA using a cellular nanoporation biochip, leading to a significant increase in PTEN mRNA accumulation within orthotopically implanted tumors and notably prolonged median survival times compared to control exosomes [253].

#### 4.1.6. Genetic Target Engineering by Fusion Expression with Glycosylphosphatidylinositol (GPI)-Anchor Signal Peptides

Glycosylphosphatidylinositol (GPI)-anchored proteins belonging to lipid raft-associated lipids were identified to be efficiently sorted and enriched in exosomes, therefore enabling exosome surface labeling through anchoring [254,255]. In 2016, anti-EGFR nanobodies were genetically fused with a GPI-anchor signal peptide, enhancing the targeting capabilities of exosomes towards EGFR-expressing tumor cells. The EGFR-specific nanobodies directed the exosomes specifically to EGFR-positive A431 cells, demonstrating that GPI-anchoring can be effectively used for displaying a range of proteins on EVs, including antibodies, reporter proteins, and signaling molecules [256].

In summary, genetic engineering of parental cells is strategically employed to upregulate components for therapeutic applications. The expression of specific cancer-targeting entities on the surface of EVs, through conjugation with membrane-associated domains such as Lamp2b, the C1C2 domain, tetraspanins, and GPI-anchor peptides, represents a promising approach for actively targeting therapeutic EVs to cancer cells and tissues.

**Table 2:** Genetically engineered exosomes for targeted anti-cancer drug delivery.

Targeting Ligand	Transmembrane Protein on EVs	Therapeutic Cargo	Cargo Loading Method	Cell Sources of EVs	Cancer Type and Targets	Function	Study Type	Year	Ref.
iRGD	LAMP-2B	DOX	Electroporation	immature dendritic cells	Breast cancer cells	Inhibit tumor growth without overt toxicity	In vitro and in vivo	2014	[99]
iRGD	LAMP-2B	DOX; GAPDH siRNA	Electroporation; Transfection	HEK293FT	Glioblastoma cells	Increase the drug internalization via across BBB	In vitro	2022	[218]
iRGD	LAMP-2B	KRAS siRNA	Transfection	HEK293T	Lung cancer cells	Target oncogenic KRAS	In vitro and in vivo	2019	[219]
iRGD	LAMP-2B	CPT1A siRNA	Transfection	HEK293T	Colon cancer cells	Target silencing CPT1A to inhibit FAO; reverse oxaliplatin resistance and inhibit tumour growth	In vitro and in vivo	2021	[220]
iRGD	LAMP-2B	BCL6 siRNA	Electroporation	immature dendritic cells	Diffuse large B-cell lymphoma cells (DLBCL)	Target silencing BLC6 to inhibit DLBCL tumor growth	In vitro and in vivo	2022	[221]
iRGD	LAMP-2B	miR-484	Electroporation	HEK293T	Ovarian cancer cells; tumor vascular endothelial cells	Inhibit angiogenesis and sensitize the cancer to chemotherapy	In vitro and in vivo	2022	[217]
tLyP-1	LAMP-2B	SOX2 siRNA	Electroporation	HEK293T	Lung cancer and cancer stem cells	Target silencing SOX2 expression of NSCLC cells and reducing the stemness of NSCLC stem cells	In vitro	2020	[224]
fragment of Interleukin 3	LAMP-2B	Imatinib; BCR-ABL siRNA	Direct incubation; Transfection	HEK293T	Chronic myeloid leukemia (CML) cells	Target delivery of Imatinib or BCR-ABL siRNA to CML cells	In vitro and in vivo	2017	[227]
HER2-binding affibody zHER	LAMP-2B	5-FU and miRNA-21 inhibitor	Electroporation	HEK293T	Her2 expressing colorectal cancer cells	Effectively reverse drug resistance and significantly enhanced the cytotoxicity in 5-FU-resistant colon cancer cells	In vitro and in vivo	2020	[183]
HER2-specific DARPin	LAMP-2B	TPD52 siRNA	Electroporation	HEK293T	HER2-positive breast cancer cells	Target silencing the TPD52 of Her2 positive cancer cells	In vitro	2019	[155]
HER2-specific DARPin	LAMP-2B	DOX	Electroporation	BM-MSCs	HER2-positive breast cancer cells	Specifically inhibit Her2 positive tumor growth	In vitro and in vivo	2019	[232]
HER2-specific DARPin	LAMP-2B	99mTc	Chemical modification	HEK293T	HER2-positive ovarian cancer cells	In vivo HER2-positive tumor imaging	In vitro and in vivo	2020	[233]
GE11/EGF	PDGFR-TM	let-7a miRNA	Transfection	HEK293T	EGFR-positive breast cancer cells	Target delivery miRNAs to EGFR expressing cancer cells	In vitro and in vivo	2013	[237]
αCD3/αEGFR	PDGFR-TM	αCD3/αEGFR	Transfection	Expi293F cells	T cell and EGFR-expressing breast cancer cells	Induce cross-linking of T cells and EGFR-expressing breast cancer cells and elicit potent antitumor immunity.	In vitro and in vivo	2018	[240]
anti-HER2 scFv antibody (ML39)	Lactadherin C1-C2 domain	CNOB and HCHrR6 mRNA	Electroporation	HEK293	HER2-overexpressing breast cancer cells	Delivery of functional exogenous mRNA to tumors	In vitro and in vivo	2018	[243]
anti-HER2 scFvs with different affinity	Lactadherin C1-C2 domain	CFSE	Chemical modification	HEK293	HER2-overexpressing cancer cells	Monitor the target delivery of antiHER2-scFvs modified exosomes	In vitro and in vivo	2018	[239]
Two copies of the HER2 ligand	Lactadherin C1-C2 domain	HER2 miRNA	Transfection	HEK293	HER2-overexpressing cancer cells	Specifically inhibit Her2 expressing tumor growth	In vitro and in vivo	2020	[244]
Anti-EGFR nanobodies	GPI-anchor Signal peptides	CellTracker Deep Red	Chemical modification	Neuro2A	EGFR-overexpressing cancer cells	Specifically target EGFR expression cells	In vitro	2016	[256]
Apo-A1	CD63	miRNA-26a	Electroporation	HEK293T	Hepatocellular Carcinoma (HepG2	Inhibit tumor cell migration and proliferation	In vitro	2018	[252]
CDX peptide/CREKA	CD47	PTEN mRNA	Cellular nanoporation biochip (CNP)	bone marrow-derived dendritic cells (BMDCs)	PTEN-deficient human U87 and mouse GL216 glioblastoma cells	Specifically inhibit PTEN-deficient glioblastoma	In vitro and in vivo	2019	[253]

### 4.2. Chemical Modification of Extracellular Vesicles

Chemical modification of isolated EVs is primarily achieved through various strategies such as conjugation reactions (including click chemistry), hydrophobic insertion, and receptor-ligand binding. These methods enhance the specificity and efficacy of EVs for targeted anti-cancer drug delivery. Examples of chemically modified EVs for targeted anti-cancer drug delivery are summarized in Table 3.

#### 4.2.1. Click Chemistry Method for Direct Modification

Chemical methods enable direct attachment of molecules to EV surfaces, with click chemistry being a particularly effective method for covalent modification of EVs under mild conditions [257]. Typically, amine groups on the exosome surface are first modified with alkyne functional groups. Subsequently, these alkyne groups can be conjugated to azido groups of targeting aptamers via azide-alkyne cycloaddition, known as ‘click’ reactions. For example, Jia et al. utilized click chemistry to attach the glioma-targeting RGE peptide (RGERPPR) to exosomes pre-loaded with superparamagnetic iron oxide nanoparticles (SPIONs) and curcumin (Cur) via electroporation. Initially, alkyne groups were conjugated to the Exo-SPION/Cur membrane through the EDC-NHS (1-ethyl-3-(3-dimethylaminopropyl) carbodiimide-N-hydroxysuccinimide) condensation reaction. Then, the RGE peptide with an azido group was attached using copper-catalyzed click chemistry, enhancing the diagnostic and therapeutic efficacy against glioma [258]. In another study, the functional alkyne groups were conjugated to the exosome surface through dibenzocyclooctyne-sulfo-N-hydroxysuccinimidyl ester (DBCO-sulfo-NHS). These were then linked to c(RGDyK) peptide (Arg-Gly-Asp-D-Tyr-Lys) equipped with an azide group via copper-free bio-orthogonal click chemistry. The resultant c(RGDyK)-modified exosomes, loaded with curcumin, showed high affinity and specificity for integrin αvβ3-expressing cells, such as human glioblastoma U87 cells and vascular endothelial cells. This modification enabled efficient BBB penetration and demonstrated the capability to suppress inflammation and cellular apoptosis in a transient middle cerebral artery occlusion mouse model [259].

#### 4.2.2. Hydrophobic Insertion Mediated Modification

Due to the hydrophobic nature of EV membranes, amphipathic molecules can be spontaneously inserted into the lipid bilayer surface of EVs, representing another chemical modification strategy—Hydrophobic insertion. Therefore, the functional molecules connected with lipophilic components in advance achieve effective incorporation into EVs through simple co-incubation [260].

*DSPE-PEG mediated self*-*assembly insertion* DSPE-PEG (polyethylene glycol (PEG)-grafted 1,2-dioleoyl-sn-glycero-3-phosphoethanolamine), an FDA-approved amphiphilic molecule for medical applications, can self-assemble into EV membrane, was successfully used to conjugate exogenous ligands for EV modification in several studies. For example, DSPE-PEG-RGD modified exosomes were used to target delivery of the vanadium carbide quantum dots (V2C QDs) as photothermal agents, enhancing the therapeutic efficiency of the anti-cancer photothermal therapy [261]. Exosomes loaded with hyaluronidase (PH20) were modified by DSPE-PEG-FA (folic acid) to produce an FA-targeted delivery system of Exos-PH20-FA. Exos-PH20-FA could efficiently reduce hyaluronidase-induced tumor metastasis. After further loading with doxorubicin (DOX), the Dox@Exos-PH20-FA system significantly improved the delivery of chemotherapy by tumor-targeting effect leveraging FA’s tumor-targeting properties [262]. Similarly, DSPE-PEG-FA modified exosomes were also applied to target delivery of ferroptosis inducer erastin to folate receptor overexpressing triple-negative breast cancer (TNBC) cells, inducing ferroptosis and reducing the proliferation and migration of MDA-MB-231 cells [207]. Additionally, aminoethylanisamide (AA), targeting ligand of sigma receptors overexpressed on lung cancer cells, was employed to modify exosomes via DSPE-PEG-AA self-assembly, enhancing drug circulation time and inhibiting pulmonary metastases [263].

*Cholesterol-mediated self*-*assembly insertion* Cholesterol is another commonly used hydrophobic moiety that facilitates the spontaneous self-assembly of various targeting ligands into exosomal membranes. For example, AS1411, a DNA aptamer with high affinity to nucleolin (which is overexpressed on the plasma membranes of breast cancer cells), was the first one to be assessed in clinical oncology trials [264]. Wang et al. applied the aptamer AS1411 covalently conjugated polypeptides with cholesterol to modify EVs for targeted delivery of let-7 miRNA/VEGF siRNA into nucleolin overexpressing breast cancer cells [265]. Similarly, AS1411 was labeled to the surface of EVs via cholesterol-PEG2000 conjugation. These AS1411-modified EVs, loaded with paclitaxel (PTX), demonstrated efficient drug delivery to target breast cancer cells both in vitro and in vivo [115]. In another study, Pi et al. engineered targeted exosomes by incorporating cholesterol conjugates with FA or RNA aptamers targeting prostate-specific membrane antigen (PSMA) and EGFR. These targeted exosomes effectively delivered survivin siRNA to specific tumor sites, significantly enhancing antitumor efficacy [266].

*Other methods mediated self*-*assembly insertion* Various hydrophobic linkers conjugated with targeting ligands have also been developed to functionalize the surface of EVs. For example, an ApoA-I mimetic peptide was conjugated to the targeting peptide LDL to facilitate EV modification by binding to the phospholipid vesicles. The resulting functionalized EVs (EVs-KLA-LDL) significantly enhanced the process of low-density lipoprotein receptor (LDLR)-mediated internalization both in vitro and in vivo, improving the delivery of the proapoptotic peptide KLA and methotrexate (MTX) to U87 glioma cells [150]. Similarly, the aptamer sgc8, which can specifically recognize membrane-expressed protein tyrosine kinase 7 (PTK7), served as a targeting ligand. utilized a diacylipid-(PEG)2 conjugated with aptamer sgc8 to modify the exosome surface. This hydrophobic interaction between the diacylipid tail and the phospholipid bilayer of the exosome provides a promising delivery platform for targeted cancer therapeutics [259].

In summary, a variety of chemical methods are employed to improve the targeting, circulation, and drug delivery capabilities of EVs, enabling further applications in diagnosis and therapy. However, the impact of these chemical modifications on the structural integrity of EVs and the biocompatibility of both the chemicals and the resulting functionalized EVs requires further evaluation.

**Table 3:** Chemically modified exosomes for targeted anti-cancer drug delivery.

Targeting Ligand	Ligand Labeling Method	Therapeutic Cargo	Cargo Loading Method	Cell Sources of EVs	Cancer Type and Targets	Function	Study Type	Year	Ref.
RGE	Copper catalyzed click chemistry	Curcumin and SPION	Electroporation	Mouse macrophage cell line Raw264.7	Glioma	Simultaneous target imaging and therapy of glioma	In vitro and in vivo	2018	[258]
c(RGDyk)	Copper free click chemistry	Curcumin	Incubation	BM-MSCs	Integrin αvβ3 overexpressing cells (U87 glioblastoma cells and vascular endothelial cells)	Increase the drug internalization via across BBB and target delivery drugs to integrin αvβ3 overexpressing cells	In vitro	2017	[259]
RGD	DSPE-PEG-RGD	V2C Quantum Dots	Electroporation	MCF-7 cells	Integrin αvβ3-poitive breast cancer MCF-7 cells	Target delivery photothermal agents to integrin expressing cells	In vitro and in vivo	2019	[261]
Folic acid (FA)	DSPE-PEG-FA	Human hyaluronidase (PH20); DOX	Transfection; Electroporation	HEK293T	Folate receptor overexpressing cancer cells	Reduce hyaluronidase-induced metastasis and enhance target delivery of chemotherapy	In vitro and in vivo	2021	[262]
Folic acid (FA)	DSPE-PEG-FA	Erastin	Sonication	Human fetal lung fibroblasts HFL-1	Folate receptor overexpressing cancer cells	Induce ferroptosis of folate receptor overexpression TNBC cells	In vitro	2019	[207]
Folic acid (FA)	DSPE-PEG-FA	DOX/P-gp siRNA	Extrusion; Electroporation	Normal ovarian epithelial Iose80 cells	Folate receptor overexpressing ovary cancer cells	Target delivery of chemotherapeutics to overcome drug resistance of ovarian can-cer	In vitro and in vivo	2023	[194]
Aminoethylanisamide (AA)	DSPE-PEG-AA	PTX	Sonication	Mouse macrophage cell line Raw264.7	Sigma receptor overexpressing lung cancer cells	Improve drug circulation and inhibit lung cancer metastases	In vitro and in vivo	2017	[263]
AS1411 aptamer	Cholesterol-polypeptides	Let-7 miRNA/VEGF siRNA	Electroporation	BMDCs	Nucleolin overexpressing breast cancer cells	Target delivery siRNAs/miRNAs to nucleolin positive cancer cells	In vitro and in vivo	2017	[265]
AS1411 aptamer	Cholesterol-PEG2000	PTX	Sonication	BMDCs	Nucleolin overexpressing breast cancer cells	Target delivery paclitaxel to nucleolin positive cancer cells	In vitro and in vivo	2018	[115]
PSMA RNA aptamer; EGFR RNA aptamer; Folic acid	Cholesterol-RNA nanoparticles	Survivin siRNA	Transfection	HEK293T	prostate cancer; breast cancer and colorectal cancer cells	Mediate RNA nanoparticles on EV memebrane	In vitro and in vivo	2017	[266]
LDL peptide	ApoA-I mimetic peptide	methotrexate, KLA (Lys-Leu-Ala)	Co-incubation	Mouse fibroblast L929 cells	LDLR overexpressing glioblastoma cells	Target treatment of LDLR overexpressing glioblastoma cells	In vitro and in vivo	2018	[150]
Aptamer sgc8	Diacylipid-(PEG)_2_	DOX	Electroporation	Immature dendritic cells (imDC)	Leukemia cells that overexpressed PTK7	Target delivery of therapeutics to PTK7 overexpressing cancer cells	In vitro	2019	[259]

## 5. Conclusion and Future Perspective

EVs have emerged as highly efficient, natural drug carriers due to their low immunogenicity, excellent biocompatibility, and high stability. This review primarily focused on the current strategies for manipulating EVs for cancer therapeutic applications, highlighting significant achievements in engineering EVs as potent therapeutic cargo delivery systems. Despite these advances, several challenges hinder their clinical translation. Firstly, EVs are harvested from diverse sources that influence their properties, yet standardization remains inadequate to ensure safety and reliability across sources and batches. Secondly, the processes of purification, characterization, and drug loading are inefficient. Developing cost-effective, large-scale production methods that allow sensitive assessment of batch-to-batch variations is imperative. Furthermore, while various techniques have been explored to enhance cargo-loading efficiency in EVs, more research is still required to improve therapeutic cargo encapsulation and develop universally applicable drug-loading methods. Thirdly, although EVs can be functionalized with exogenous materials to enhance targeting and circulation properties, the potential immunogenicity and disruption of EV membrane properties by these modifications necessitate further investigation to ensure the development of safe and effective drug delivery systems.

A deeper understanding of EV properties and functions could revolutionize their use as long-term, safe, and efficient drug delivery platforms. The complex composition of EVs, including constituents that may promote cancer, underscores the need for rigorous quantification of carcinogenic components and the establishment of strict standards for these substances before clinical use. Additionally, the unique membrane structure of EVs provides significant advantages over synthetic lipid nanostructures for drug delivery. However, methods to remove internal contents while preserving biophysical and functional properties for efficient drug loading need further development. Moreover, there is a need for a comprehensive exploration of EV membrane components. Research has largely focused on the roles of the contents in cancer progression, with less attention paid to the composition and function of EV membranes. Identifying various EV membrane components and their specific functions will not only enhance our understanding of their roles in cancer progression but also optimize their structures to improve delivery efficiency and minimize potential side effects. This insight will also aid in distinguishing EVs from different sources and facilitating the development of more effective synthetic nano-delivery systems.

Overall, EVs offer new perspectives on the efficient delivery of therapeutic agents for cancer treatment. Their nano-scale properties and inherent biocompatibility position EVs as a particularly promising platform for targeted therapy. With continued research efforts, the utilization of EVs as a targeted delivery platform holds great promise for the future of targeted cancer therapies.
